# Results of a New Technique for Implantation of Nonrestrictive Glaucoma Devices

**DOI:** 10.5005/jp-journals-10008-1151

**Published:** 2013-09-06

**Authors:** Gabriel Enrique Ortiz Arismendi, Cristina Del Pilar Peña Valderrama, Oscar Albis-Donado

**Affiliations:** Associate Professor and Chief of Glaucoma Section, Ophthalmology Unit Faculty of Medicine, Universidad Nacional de Colombia, Clinica de Ojas Bogota, DC; Assistant Professor, Ophthalmology Unit, Faculty of Medicine Universidad Nacional de Colombia, Bogotá, DC, Colombia; Professor, Department of Ophthalmology, Association Para Evitar la Ceguera en Mexico, Mexico City, Mexico

**Keywords:** Glaucoma surgery, Glaucoma implants, Titrated ligature.

## Abstract

**Objective:** To describe and present results of an original technique for nonvalved glaucoma implants.

**Patients and methods:** Thirty-five eyes of 34 patients with aggressive and/or advanced glaucomas of different causes were included. A Baerveldt implant was used in all cases, using an absorbable ligature that had been titrated to allow fow from day 1, but avoiding hypotony. Intraocular pressure (IOP) during the first 8 weeks, final IOP, visual acuity and complications were analyzed.

**Results:** Mean preoperative IOP was 42.8 mm Hg (range: 24-64 mm Hg). IOP was 14.4, 17.2, 18.6, 19 and 16.4 mm Hg during the 1, 2, 4, 6 and 8 postoperative weeks. Mean final IOP was 13.8 ± 4.25 mm Hg, a 67.8% reduction, after a mean follow-up time of 13 months (range: 8-29 months). Twenty-nine eyes (82.9%) had complete success, two had qualifed success (5.7%) and four were failures (11.4%). Choroidal detachments and transient tube obstructions were the most frequent complications.

**Conclusion:** Titrated ligature of Baerveldt tubes was effective for controlling IOP during both the early and late postoperative phases in eyes with severe glaucomas.

**How to cite this article:** Arismendi GEO, del Pilar Peña Valderrama C, Albis-Donado O. Results of a New Technique for Implantation of Nonrestrictive Glaucoma Devices. J Current Glau Prac 2013;7(3):130-135.

## INTRODUCTION

Glaucoma implants are a valuable alternative for controlling intraocular pressure (IOP) in difficult to treat glaucomas that have been in use for the past 30 years. All implants share some common features, although design, materials and size differ. In all a tube is inserted into the anterior chamber that is connected to a main body or plate, located at or behind the equator, around which a fibrous capsule that regulates aqueous outfow is formed. Implants may have restrictive fow (e.g. Ahmed or Krupin) or unrestrictive (e.g. Molteno and Baerveldt).^1^

Implants with restrictive fow are designed to permit a more controlled flow from the beginning, while the tube must be occluded fully or partially during the first postoperative days in unrestricted implants to avoid severe hypotony. Occluding the tube will raise IOP until the ligature is removed or reabsorbed, an event that is usually planned for a time when sufficient fibrosis is formed around the implant, usually after 3 to 6 weeks. During this hypertensive period, eyes with advanced glaucomas or those with very high initial IOP might suffer additional devastating damage. Making venting slits anterior to the ligature, or using a suture inside the tube to permit some limited fow are established techniques that tend to have unpredictable results.^2,3^

After years of using Baerveldt implants we have devised the ‘Ortiz' partial titrated ligature' technique to lower IOP from the first postoperative day with limited fow that lasts until the implant begins functioning fully.

## PATIENTS AND METHODS

We report a case series of patients with difficult to control glaucomas, in whom a Baerveldt implant with partial titrated ligature was performed. Eyes in immediate need of an implant, with very high IOP on maximum tolerated therapy and/or advanced glaucoma damage were included. We excluded patients unable to comply with visits and those with less than 6 months of follow-up.

A signed informed consent was obtained from each patient after explaining the nature of the study and of the procedure. Our hospital's surgery investigation department and the ethics committee of the faculty of medicine of the Universidad Nacional de Colombia approved the protocol. Data was collected in a specially designed form that registered age, sex, race, diagnosis, date of the surgery, type of procedure, implant location and tube location, initial and final visual acuity, complications, additional procedures needed and initial IOP, plus IOP at day 1, weeks 1, 2, 4, 6 and 8th, and last recorded IOP.

Complete success was defined as an IOP above 5 and under 22, with an IOP reduction of 30% or better without glaucoma medications, and qualifed success if they needed glaucoma medications to reach this goal. Eyes with failure had IOP fewer than 6, above 21, less than 30% reduction, loss of light perception or phthisis bulbi. Reinterventions such as anterior tube revisions, YAG laser to unocclude the tube, or repair of an exposed tube were not considered failures as long as other criteria were still met.

**Fig. 1:**
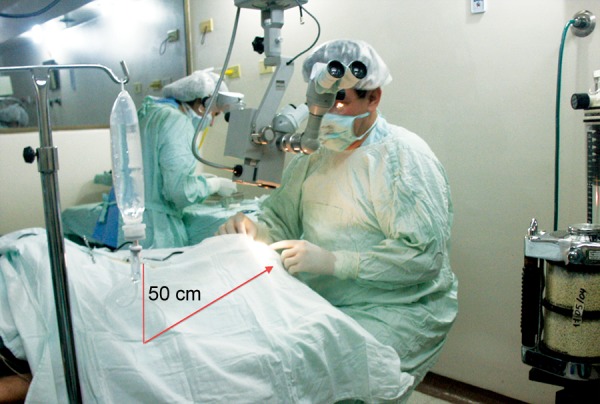
The height of the irrigation bottle was 50 cm above the level of the eye that needed to be operated, a column that corresponds to approximately 30 mm Hg for doing the ligature titration

**Fig. 2:**
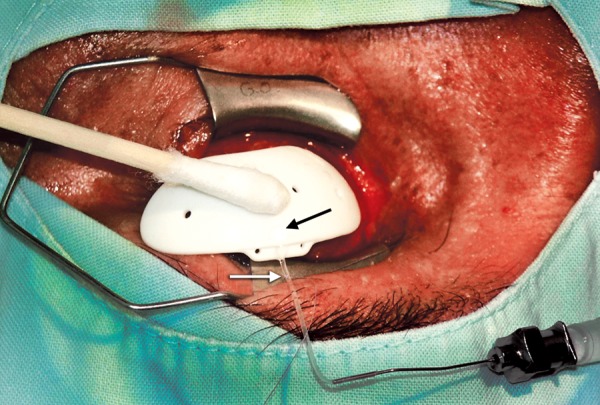
The tubing connects the irrigation bottle to the tube of the implant via a 26G cannula and permeability is checked. The tube is ligated with an absorbable suture (white arrow) and the tension is adjusted until a very slow fow is achieved before the knot is secured (dark arrow)

## SURGICAL PROCEDURE

The tube of the implant was connected through a 26G cannula to a BSS bag placed 50 cm above the head of the patient. Every 15 cm of height above the eye represents about 10 mm Hg, so any fow that occurred at this level would mean that pressure would be less than 30 mm Hg (Fig. 1). Once the tube was tested for permeability the tube was ligated with an absorbable suture (7-0 or 8-0 polyglactin, Vicryl^®^ Ethicon Inc.) titrating for a slow, continuous fow of BSS, similar to checking the fow of a trabeculectomy (Fig. 2). Once the desired fow was obtained the suture was locked in place with 5 knots. Then the implant was fixed in place in the conventional manner, in the superotemporal quadrant, using a long-needle tract with a 23G needle bent as a Z without a patch (Dr Felix Gil's Technique).^4^

### Postoperative Regimen

Every patient was examined on the first or second day, and at the end of weeks 1, 2, 4, 6 and 8 as per protocol, then every 2 to 3 months depending on IOP behavior or complications behavior. A topical antibiotic was used during the first week and prednisolone 1% every 2 to 4 hours during 8 to 10 weeks. Whenever, hypotony was present prednisolone was replaced with a nonsteroidal anti-inflammatory to promote a greater and faster scarring around the implant.

## RESULTS

During the study period (January 2000 to December 2003) 53 eyes (19 were left eyes) of 51 patients received a Baerveldt implant, but 18 had to be excluded due to a short follow-up period.

We included 35 eyes of 34 patients, of which 22 were women; mean age was 59.9 years (range 22-73), including 30 mestizos, 3 Caucasian and 1 black. Demographic and surgical data for each case are included in Table 1. Most cases (25) received a Baerveldt 350 mm^2^ implant and the rest (10) a 425 mm^2^ implant. The tube was inserted into the anterior chamber in most eyes, 6 eyes additionally required an anterior or pars plana vitrectomy to avoid tube blockage.

Visual acuity improved in at least 1 line in 9 eyes, remained the same in 19 and worsened in 7. Initial visual acuity ranged from LP to 20/60 and final visual acuity from NLP to 20/40 (Table 2).

During follow-up the tube got blocked with vitreous in five cases, one could be treated with YAG laser vitreolysis, another also required medications and the other three additional surgical vitrectomy; one of these ultimately failed. One tube retracted out of the anterior chamber and needed to be relocated without loss of IOP control. Seven cases had transient hypotony with no choroidal detachments; only one of them had a mild shallow anterior chamber. Four additional cases had choroidal detachments, three of which were solved spontaneously; the other had to be drained. A patient who initially had light perception only had repeated tube extrusions and after finding calcifed material inside the lumen, the tube was trimmed and removed from the anterior chamber. It was considered a failure and eventually needed cyclocryotherapy to further control IOP. In total 13 eyes needed additional procedures to either free the tube or relocate it in a better position (Table 3).

**Graph 1:**
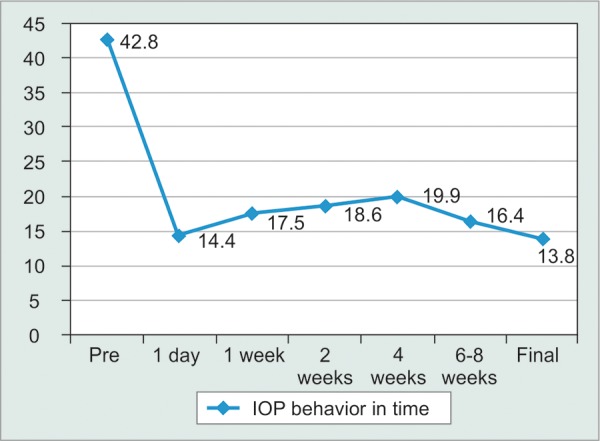
IOP behavior (in mm Hg) from the preoperative IOP, during the first 8 postoperative weeks and at last visit

Mean initial IOP was 42.8 mm Hg (range: 24-64), and it was 14.4, 17.2, 18.6, 19 and 16.4 mm Hg during the 1, 2, 4, 6 and 8th postoperative weeks. Mean final IOP was 13.8 ± 4.25 mm Hg, a 67.8% reduction, after a mean follow-up time of 13 months (range: 8-29 months, Table 2, Graph 1).

**Graph 2:**
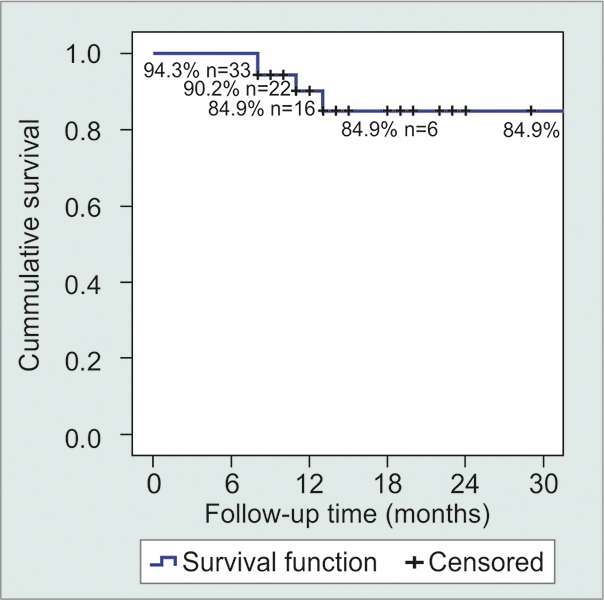
Kaplan-Meier survival plot, n indicates the number of remaining successful cases at each time-point

Mean IOP for 425 mm^2^ implants was 14.7 mm Hg and it was 13.5 mm Hg for the 350 mm^2^ implant, a nonsignificant difference. Twenty-nine eyes (82.9%) had complete success, two had qualifed success (5.7%) and four were failures (11.4%, Table 2, Graph 2). Two failures were due to tube obstruction with vitreous, one to repeated tube extrusion and one neovascular glaucoma that went from hand movements to no light perception.

**Table 1:** **Table 1:** Patients, diagnosis and procedures

*No.*		*Age*		*Sex*		*Glaucoma diagnosis*		*Race*		*Tube location*		*Implant*		*Ligature material*	
1		54		M		Pseudophakic AC IOL		MES		PC		Baerveldt 425		7-0 Vicryl	
2		62		M		Pseudophakic		MES		AC		Baerveldt 350		7-0 Vicryl	
3		75		F		PK		MES		AC		Baerveldt 350		7-0 Vicryl	
4		76		F		CACG		MES		AC		Baerveldt 350		7-0 Vicryl	
5		50		M		PK		MES		AC		Baerveldt 350		7-0 Vicryl	
6		72		F		PK		MES		PC		Baerveldt 425		7-0 Vicryl	
7		65		F		Uveitic		CAU		AC		Baerveldt 350		7-0 Vicryl	
8		65		F		Uveitic		CAU		AC		Baerveldt 350		7-0 Vicryl	
9		61		F		Pseudophakic		MES		VC		Baerveldt 350		7-0 Vicryl	
10		70		F		Uveitic		MES		AC		Baerveldt 350		7-0 Vicryl	
11		69		F		PK		BLA		VC		Baerveldt 350		7-0 Vicryl	
12		65		F		Aphakic		MES		VC		Baerveldt 350		8-0 Vicryl	
13		49		M		Pseudophakic		MES		AC		Baerveldt 350		7-0 Vicryl	
14		43		F		Pseudophakic		MES		AC		Baerveldt 350		7-0 Vicryl	
15		49		M		Pseudophakic		MES		AC		Baerveldt 350		7-0 Vicryl	
16		38		M		Uveitic		CAU		AC		Baerveldt 350		7-0 Vicryl	
17		69		F		PK		MES		PC		Baerveldt 350		7-0 Vicryl	
18		35		M		NVG		MES		AC		Baerveldt 350		7-0 Vicryl	
19		65		M		Pseudophakic		MES		AC		Baerveldt 350		7-0 Vicryl	
20		60		F		GPAA		BLA		AC		Baerveldt 350		7-0 Vicryl	
21		60		M		PK		MES		AC		Baerveldt 425		7-0 Vicryl	
22		72		F		PK		MES		AC		Baerveldt 425		8-0 Vicryl	
23		64		F		Pseudophakic		MES		PC		Baerveldt 425		7-0 Vicryl	
24		72		F		Pseudophakic		MES		VC		Baerveldt 350		7-0 Vicryl	
25		37		F		PK		MES		PC		Baerveldt 350		7-0 Vicryl	
26		67		F		PK		MES		AC		Baerveldt 350		7-0 Vicryl	
27		22		F		PK		MES		AC		Baerveldt 425		7-0 Vicryl	
28		65		F		PK		MES		PC		Baerveldt 425		7-0 Vicryl	
29		38		M		Post-traumatic		MES		AC		Baerveldt 350		7-0 Vicryl	
30		62		M		PK		MES		AC		Baerveldt 425		7-0 Vicryl	
31		67		F		Pseudophakic		MES		AC		Baerveldt 350		7-0 Vicryl	
32		73		F		PK		MES		AC		Baerveldt 425		7-0 Vicryl	
33		64		M		PK		MES		AC		Baerveldt 350		7-0 Vicryl	
34		70		F		PK		MES		PC		Baerveldt 350		7-0 Vicryl	
35		73		F		Pseudophakic		MES		AC		Baerveldt 425		7-0 Vicryl	

AC: Anterior chamber; PC: Posterior chamber; VC: Vitreous cavity; MES: Mestizo; BLA: Black; CAU: Caucasian; IOL: Intraocular lens; PK: Penetrating keratoplasty; M: Male; F: Female; CACG: Chronic angle closure glaucoma; POAG: Primary open angle glaucoma; NVG: Neovascular glaucoma

## DISCUSSION

We found that modifying Baerveldt implant technique by using ‘Ortiz' partial titrated ligature' was useful in our group of patients to lower IOP significantly during the immediate postoperative period, although not all eyes to normal levels. Long-term results are also encouraging, since our success rate was 88.6% (84.9% cumulative success rate at 30 months by Kaplan-Meier), while maintaining a low complications rate, many transient and not needing many reinterventions. In fact, the seven cases of transient hypotony and the three cases of transient choroidal detachments are comparable to the rates of the same complications reported for restrictive implants or even for unrestricted implants with full ligature after it has dissolved.^5,7^

**Table 2:** **Table 2:** IOP, visual acuity and final result

		*Visual acuity*				*Intraocular pressure (mm Hg)*															
*No.*		*Preoperative*		*Final*		*Pre*		*1 day*		*1 week*		2 *weeks*		*4 weeks*		*6-8 weeks*		*Final*		*Result*	
1		HM		HM		48		24		30		34		38		26		16		Success	
2		20-60		20-40		41		18		19		17		18		12		17		Success	
3		HM		HM		32		7		17		23		22		13		13		Success	
4		CF1M		CF 1M		38		9		17		24		3		8		8		Success	
5		CF 50 cm		HM		40		4		12		19		20		14		12		Success	
6		HM		HM		66		16		24		30		54		9		9		Success	
7		20/100		20/70		31		15		8		7		7		8		12		Success	
8		CF		CF		28		5		18		17		6		15		15		Success	
9		HM		HM		66		16		10		11		12		10		14		Success	
10		CF 20 cm		CF 20 cm		30		5		4		4		7		15		9		Success	
11		LP		LP		50		35		30		26		17		16		17		Success	
12		HM		HM		38		2		2		14		14		13		18		Success	
13		CFA1M		CF2M		28		19		21		20		25		24		23		Failure	
14		HM		HM		39		9		12		14		—		8		13		Success	
15		LPP		HM		29		23		9		10		12		10		12		Success	
16		20/400		20/400		28		4		7		7		4		16		12		Success	
17		HM		CF2.5M		52		28		24		28		29		22		20		Relative success	
18		HM		NLP		60		18		38		30		10		39		15		Failure	
19		CF 50 cm		CF 50 cm		44		22		23		20		22		15		17		Success	
20		20/60		20/200		24		22		24		—		28		10		14		Success	
21		HM		CF 20 cm		46		10		9		11		13		14		16		Success	
22		HM		HM		41		5		6		15		10		31		17		Success	
23		HM		NLP		60		16		30		22		30		19		24		Failure	
24		20/400		20/400		32		2		9		9		6		6		10		Success	
25		LP		LP		59		17		16		—		15		15		14		Success	
26		HM		HM		32		7		27		34		35		40		12		Success	
27		CF 50 cm		CF 50 cm		56		7		12		12		11		12		10		Success	
28		HM		CF 1M		46		20		26		22		46		22		8		Success	
29		20/100		20/300		40		21		25		24		11		12		11		Success	
30		CF3M		CF3M		39		17		16		14		18		12		16		Success	
31		CF2M		20/800		28		22		20		23		20		14		13		Success	
32		CF 50 cm		CF2M		55		18		18		15		13		15		13		Success	
33		HM		LP		46		32		26		28		29		11		4		Failure	
34		CF 20 cm		CF 20 cm		42		8		7		16		59		29		12		Success	
35		LPP		LPP		64		2		16		15		12		20		18		Relative success	
Mean						42.8		14.429		17.235		18.63		19.6		16.42		13.82			

NLP: No light perception; LP: Light perception; LPP: Light perception and projection; HM: Hand movements; CF: Counts fingers

**Table 3:** **Table 3:** List of complications, some eyes had more than one

*Complications*		*n*		*Total (%)*	
Choroidal detachment		4		11.4	
Tube occlusion		4		11.4	
Tube exposure		4		11.4	
Hyphema		2		5.7	
Hypotony		1		2.9	
Flat anterior chamber		1		2.9	
Tube migration		1		2.9	
Tube extrusion		1		2.9	
Vitreous hemorrhage		1		2.9	
Endothelial contact		1		2.9	
Uveitis		1		2.9	

Studies comparing restrictive and unrestricted implants have shown variable results.^8,9^ A previous comparison of Ahmed *vs* Baerveldt 350 that used similar success criteria as our study, found similar results between them, with final IOP of 12.1 ± 5.3 mm Hg and 13.6 ± 5.6 mm Hg, but complete success rates of 15.6 and 18.7%, plus qualified success rates of 50 and 46.8% were not as good as in our series. The rates of hypotony were 34.4% for the Ahmed and 37.5% for the Baerveldt implants with venting slits in some eyes.^10^ We observed seven cases (20%) of early hypotony with spontaneous resolution during the first 2 weeks in 6, and at week 6 in the other.

Shallow anterior chamber was also present in one eye (2.8%), a better rate than the typical 5 to 44% reported with several other implants.^6-9^

A hypertensive phase has been reported in up to 60% of Ahmed valves, beginning between weeks 2 and 6, requiring antiglaucoma medications and that will get better in a small percentage of cases after several months.^11^ A more recent study showed that the hypertensive phase lasted more than a year despite the use of mitomycin C, in 40% of cases if a partial removal of Tenon's was performed and in 46% when it was not done.^12^ This prolonged hypertensive period was not present in our series, although a mild IOP elevation did occur in 4 eyes between months 2 and 3 that was spontaneously solved. Another 3 cases had early IOP elevation due to a too tight ligature or tube obstruction with either fibrin or vitreous.

Experimental studies in animals and humans have shown the formation of a fibrous capsule around the plate, which is responsible for primary resistance to aqueous outfow, and is made up of an inner acellular collagen band with spaces among its strands, an intermediate layer with greater organization and an external vascularized layer.^14^

There are three different Baerveldt models, with surface areas ranging from 250, 350 and 425 mm^2^. The surface area for the Ahmed valve is 185 mm^2^ (and also of each additional plate), it is 184 mm^2^ for the Krupin and each Molteno plate has an area of 134 mm^2^. Several studies have found that a greater surface area is related with a better long-term aqueous outfow and a lower IOP, supporting a size of around 268 mm^2^ for a Molteno-type implant and 350 mm^2^ for Baerveldt.^13-15^ The roleof aqueous in the bleb during the initial postoperative period might decrease fibrosis and be related to better IOP in the long run, so having some fow of aqueous initially might be desirable.^14,16^ This is an advantage of restricted implants and might explain why IOP reduction might be similar despite their smaller size and lower long-term outfow.

Among the disadvantages of restricted implants a higher risk of obstruction with detritus or infammatory cells that might predispose them to a higher risk of a hypertensive phase.

An implant that is closer to the ideal should have a larger area for long-term IOP control, a good aqueous outfow that will indefinably keep those IOP levels without peaks, but with a low risk of hypotony despite having an effective IOP lowering in the early postoperative period.

Our method is in line with all these postulations, but requires experience and is affected by the subjectivity of the surgeon during fow titration, which makes it less reproducible. A more exact and standardized method to restrict early fow during the first weeks that eventually frees full fow is needed.

## SUMMARY

The modifed surgical technique that we used in this group of patients, allowed us to obtain a success rate that compares favorably with most published studies on glaucoma implants. Further studies to ascertain the reproducibility of the technique, the results and even the design of new implants aiming to improve long-term results and reduce complications are needed.
